# Advances in Wood-Based Composites

**DOI:** 10.3390/polym17081104

**Published:** 2025-04-18

**Authors:** Lubos Kristak, Roman Reh, Marius Catalin Barbu, Eugenia Mariana Tudor

**Affiliations:** 1 Faculty of Wood Sciences and Technology, Technical University in Zvolen, T. G. Masaryka 24, SK-960 01 Zvolen, Slovakia; reh@tuzvo.sk; 2 Forest Products Technology and Timber Construction Department, Salzburg University of Applied Sciences, Markt 136 a, 5431 Kuchl, Austria; marius.barbu@fh-salzburg.ac.at (M.C.B.); eugenia.tudor@fh-salzburg.ac.at (E.M.T.); 3 Faculty of Furniture Design and Wood Engineering, Transylvania University of Brasov, B-dul. Eroilor nr. 29, 500036 Brasov, Romania

## 1. Introduction

The significance of wood-based composites has grown substantially in recent years due to their enhanced material efficiency, sustainability, and versatile applications. These composites are engineered by integrating lignocellulosic wood elements, such as fibers, particles, or veneers, with thermoplastic or thermosetting polymeric adhesives through various manufacturing processes, including hot pressing, extrusion, or injection molding. Recent advancements in wood-based composites have focused on multiple facets, including processing techniques, product innovations, surface and color treatments, and the examination of key properties. Furthermore, bio-based layered hybrid composites are being researched to provide sustainable alternatives in structural applications, alongside a focus on diversifying the sourcing of raw materials. To optimize performance characteristics, specific additives such as hardeners, coupling agents, plasticizers, and fillers are incorporated into the composite formulation. These additives play a critical role in improving interfacial adhesion, mechanical strength, dimensional stability, and resistance to environmental degradation, thereby enhancing the overall durability and functionality of the material. Additionally, energy efficiency is critical; therefore, improved manufacturing effectiveness and sustainability are targeted. Finally, significant insights have been gained into the acoustic properties of wood through the analysis of sound propagation velocity, combined with impacting design choices. In addition, the evaluation of cellulose macromolecular properties after chemical treatment helps us understand the changes that affect the performance of wood composites, indicating a comprehensive approach to enhancing wood-based materials.

To optimize the structural integrity and performance of wood-based composites, specific additives are incorporated into their formulation. These additives include hardeners, coupling agents, plasticizers, flame retardants, fungicides, and fillers, each serving unique roles in modifying the physical and chemical properties of the polymer matrix [1,2,3,4]. Hardeners improve curing efficiency, leading to superior bonding strength, while coupling agents enhance interfacial adhesion between hydrophilic wood components and hydrophobic polymer matrices. Plasticizers increase flexibility, reducing brittleness, whereas fillers such as calcium carbonate, talc, or nanocellulose enhance dimensional stability and mechanical resilience. Additionally, flame retardants and fungicides improve fire resistance and biological durability, respectively, extending the material’s service life in demanding environments [5,6,7,8,9]. Wood-based composites have gained widespread acceptance in both structural and non-structural applications, making them integral to various industries. In the construction sector, they are used for load-bearing and non-load-bearing elements such as beams, wall panels, sheathing, and formwork due to their high strength-to-weight ratio and resistance to warping. In interior applications, they are commonly utilized in furniture manufacturing, cabinetry, flooring, decorative panels, and moldings, offering aesthetic appeal combined with functional benefits [10,11,12,13,14]. Exterior applications include decking, fencing, cladding, and doors, where enhanced weather resistance and UV stability are critical factors. The automotive and aerospace industries also incorporate wood–plastic composites in lightweight, durable components to improve fuel efficiency and reduce environmental impact [15]. Advancements in wood-based composite technology continue to drive improvements in mechanical performance, moisture resistance, biodegradability, and recyclability. Emerging trends focus on the development of bio-based and eco-friendly adhesives derived from renewable resources, reducing reliance on synthetic petrochemical-based binders. Additionally, nanotechnology and fiber modification techniques are being explored to further enhance composite strength, thermal stability, and fire resistance. With increasing emphasis on sustainable materials and circular economic principles, wood-based composites are expected to play a crucial role in reducing deforestation, minimizing waste, and promoting green construction practices in the coming decades [16,17,18]. One of the ways to reduce deforestation is to use alternative raw materials from which composites can be made [19,20,21,22,23,24,25]. In the case of particleboards, a promising method is the use of alternative, lesser-known wood species in particleboard production.

## 2. An Overview of Published Articles

Reh et al. (contribution 1) analyzed perspectives on using alder, larch, and birch wood species to maintain the increasing particleboard production flow. These three wood species represent an eco-friendly and sustainable wood alternative to the conventional wood raw materials used. This review confirms the diversity of the use of these three species in different fields and proves their suitability for particleboard production.

Wood-based composites have gained significant attention in the global market, necessitating a deeper understanding of their physical and mechanical behavior, particularly in relation to adhesive polymerization. Silva et al. (contribution 2) investigated the effects of incorporating aluminum oxide and aluminum oxide copper nanoparticles into a urea-formaldehyde-based polymeric adhesive using an environmentally friendly approach. The manufactured *Eucalyptus urophylla* var. grandis wood composites were analyzed for their resin properties such as viscosity, gel time, and pH, as well as panel characteristics including density, moisture content, thickness swelling, modulus of elasticity, modulus of rupture, and thermal conductivity. The results showed that nanoparticle addition influenced viscosity, while all treatments exhibited a basic pH. However, gel time could not be determined after 10 min. No significant improvement was observed in swelling resistance, density, or moisture content. An increase in pressing temperature from 150 °C to 180 °C positively affected all physical and mechanical properties, suggesting enhanced polymerization of the adhesive. Overall, the incorporation of 0.5% nanoparticles had a limited effect on improving the physical–mechanical performance of the particleboards.

The modification of urea-formaldehyde adhesives involves altering its chemical composition or incorporating additives to enhance its performance in terms of bonding strength, durability, moisture resistance, and environmental impact, the last of which was the goal of the research by Reh et al. (contribution 3). The potential of using ground beech (*Fagus sylvatica* L.) bark as an eco-friendly additive in UF adhesives for molded plywood manufacturing was investigated. Nine-layered, flat, and molded plywood was produced under industrial conditions from beech veneers bonded with a UF adhesive mixture. The positive effect of beech bark in the UF adhesive mixture on a reduction in formaldehyde emissions from the molded plywood was also confirmed. Beech bark, considered to be wood-processing industry waste or a byproduct, has significant potential to be used as a filler in UF resins for molded plywood production, providing an environmentally friendly, inexpensive solution for the industrial valorization of bark as a bio-based formaldehyde scavenger.

The ongoing advancements in science and technology are driving a growing demand for environmentally friendly products derived from natural sources, as well as the enhanced reutilization of forestry and agricultural byproducts, which are often regarded as waste. The use of polymer blends to bond fibers for wood-based products seems an attractive substituent because of the low cost and suitability for a wide range of applications. Natural fiber-reinforced polymers are increasingly replacing synthetic fiber-reinforced plastics across various industrial sectors, including automotive manufacturing, packaging, and furniture production. These bio-based composites offer reduced weight and enhanced thermal properties, contributing to improved sustainability and performance. Gumowska et al. (contribution 4) elaborated on layered composites produced with different biopolymer adhesive layers, including biopolymer polylactic acid, polycaprolactone, and biopolymer blends of PLA + polyhydroxybutyrate with the addition of microcrystalline cellulose and triethyl citrate for these blends, which acted as binders and co-created the five layers in the elaborated composites. The modulus of rupture, modulus of elasticity, internal bonding strength, and density profile were obtained, and differential scanning calorimetry, thermogravimetric analysis, and scanning electron microscopy analysis were performed. The results showed that among the composites in which two pure biopolymers were used, PLA obtained the best results. The results of this study demonstrate the feasibility of producing layered wood-based composites using various biopolymers and their blends as specialized property layers and binders. This approach enables the development of formaldehyde-free wood-based composites that enhance the inherent properties of both wood and biopolymers, offering improved performance and sustainability.

The physicochemical and mechanical properties of wood-based materials can be significantly improved through impregnation, a process in which functional substances penetrate the wood’s cellular structure, modifying its intrinsic characteristics. Impregnation involves deep penetration of resins, chemicals, or nanoparticles into the wood structures. Rahayu et al. (contribution 5) used TiO_2_ nanoparticle impregnation and analyzed their effect on the density and dimensional stability of mangium wood and the effectiveness of the presence of the impregnation in wood in degrading pollutants. The samples were analyzed for density, weight percent gain (WPG), and bulking effect (BE). The samples were also analyzed via X-ray diffraction (XRD) and Fourier-transform infrared spectroscopy (FTIR). TiO_2_ nanoparticles resulted in an increase in density, WPG, and BE-treated mangium. Based on the XRD and FTIR results, the TiO_2_ nanoparticle was successfully impregnated into mangium wood. A scanning electron microscopy–energy-dispersive X-ray spectroscopy analysis indicated that TiO_2_ nanoparticles covered the surface of the wood cells. The TiO_2_-impregnated mangium wood has a higher photocatalyst activity than the untreated wood, indicating better protection from UV radiation and pollutants.

Wood preservation involves various methods to extend its lifespan and protect it from decay, insects, mold, and weathering. The effect of using selected inorganic chemicals as the main components of waterborne wood preservative systems on the degradation of the cellulose constituent in wood from model samples was examined by Jurczykova et al. (contribution 6). Whatman papers, as pure cellulose model samples, were impregnated with 10 different 5 wt% solutions of inorganic salts and distilled water and consequently subjected to wet-thermal accelerated aging. The samples were then derivatized to cellulose tricarbanilates through two different procedures (by precipitation in a methanol–water mixture/by evaporation of pyridine from the reaction mixture) and finally analyzed using size exclusion chromatography. Chemically treated and aged cellulose samples showed different changes in their degree of polymerization and polydispersity in terms of untreated non-aged standards caused by different ongoing degradation reactions, such as dehydration, hydrolysis, oxidation, and cross-linking. This paper brings new insights regarding the complex evaluation of the polymeric properties of degraded cellulose by considering all important factors affecting the sample and the analysis itself through the use of statistics.

Thermal modification is another environmentally friendly process that enhances the durability, stability, and resistance of wood by exposing it to elevated temperatures, mostly in the absence of oxygen. This treatment alters the wood’s chemical structure, reducing its hygroscopicity and improving its resistance to biological degradation. Jurczkyova et al. (contribution 7) examined the effect of thermal modification temperature on the selected optical properties of six tropical wood species—Sp. cedar (*Cedrala odorata*), iroko (*Chlorophora excelsa*), merbau (*Intsia* spp.), meranti (*Shorea* spp.), padouk (*Pterocarpus soyauxii*), and teak (*Tectona grandis*). The CIELAB color space parameters, yellowness, ISO brightness, and UV-Vis diffuse reflectance spectra were obtained. Subsequently, these wood samples were extracted into three individual solvents (acetone, ethanol, and ethanol-toluene). The yields of the extracted compounds; their absorption spectra; and again, their L*, a*, b*, DE*, and Yi parameters were determined. The results showed that thermal modification above 200 °C causes tropical wood to lose its characteristic color, which contributes to the unique aesthetic appeal of individual species. Although the process results in a more homogeneous appearance, it significantly reduces the wood’s brightness and yellowness. At temperatures above 220 °C, color differences become negligible, as confirmed by a cluster analysis within the PCA (Principal Component Analysis). This uniformity may not always be desirable, as some consumers select wood primarily for its decorative properties. Ultimately, the suitability of thermally modified wood depends on its intended application and whether color consistency is a priority or if other advantages of ThermoWood, such as enhanced durability and stability, take precedence.

Fine art coatings are typically formulated by combining metal fillers with water-based coatings, which are then applied to the surfaces of wood structures, furniture, and crafts for decorative purposes. However, their durability is often constrained by limited mechanical strength. In contrast, the dispersion of metal fillers and the mechanical performance of the coating can be significantly enhanced through the use of coupling agents, which facilitate strong interfacial bonding between the resin matrix and the metal filler. Han and Yan (contribution 8) examined the effect of silane coupling agent modification and its influence on a brass powder–water-based acrylic coating. Three different silane coupling agents, 3-aminopropyltriethoxysilane, -(2,3-epoxypropoxy)propytrimethoxysilane, and -methacryloxypropyltrimethoxysilane, were used to modify the brass powder filler in orthogonal tests. The artistic effect and optical properties of the modified art coating induced by different proportions of brass powder, silane coupling agents, and pH were compared. The results demonstrated that the amount of brass powder and the kind of coupling agent used had a substantial impact on the coating’s optical characteristics. These results provide a technological foundation for the development of artistic coatings for wood.

The influence of coating treatments on sound propagation speeds in thin boards made of spruce and maple resonance wood was examined by Faktorova et al. (contribution 9). Wood samples, both unvarnished and coated with oil-based or alcohol varnish, were exposed to UV radiation and saline fog. Lamb wave propagation was analyzed using a semicircular test model to assess acoustic responses in the longitudinal and radial directions. A statistical analysis revealed that oil-based varnish reduced propagation speed mainly in the radial direction, while alcohol varnish had a greater effect longitudinally in spruce wood. In maple wood, increasing varnish layers decreased the anisotropy ratio between the longitudinal and radial directions, regardless of varnish type. Research showed that surface treatments applied to wooden resonance plates affect sound propagation speeds in both the longitudinal and radial directions. The acoustic behavior of the wood is influenced by both the type of treatment and the wood’s anatomical characteristics, with variations in propagation speeds observed even among samples of the same species due to differences in their anatomical structure.

In furniture production, efforts are being made to lower energy consumption while adopting green practices. These include using sustainable materials, optimizing manufacturing processes, and implementing energy-efficient technologies. By reducing energy use and minimizing environmental impact, the industry can produce eco-friendly furniture that meets both consumer demands and sustainability goals. Pakula et al. (contribution 10) analyzed the support of eco-design ideas and sustainable manufacturing techniques by examining the energy consumption related to drilling holes for various furniture connections such as eccentric joints, confirmat screws, and dowels. The energy consumption was measured using a portable power quality analyzer. The measurement process involved recording energy consumption at different stages of the machining process, allowing for an analysis of specific cutting work and total energy consumption for various joint types. The results indicate that connecting furniture with dowels consumes the least energy but is the least user-friendly, as it does not allow for disassembly, reassembly, or easy transport. While eccentric joints consume more energy than dowels, they offer greater convenience for transportation and easier assembly. However, each time the furniture is reassembled, it loses strength due to material tearing caused by the removal of the confirmat screw. The highest energy consumption for drilling holes is associated with the eccentric joint, which, despite requiring more energy and larger holes, provides the best durability, ease of assembly, and ability to disassemble and transport the furniture without compromising its strength. Ultimately, it can be concluded that enhancing the usability of furniture increases the use of technology and, consequently, energy consumption. This study offers valuable insights into energy consumption associated with different furniture joints, providing critical information for eco-design and sustainable manufacturing practices. The analysis highlights the varying energy demands of each joint type, which can inform the development of more energy-efficient and environmentally friendly methods for furniture production.

## 3. Conclusions

The collective contributions in this compilation reflect a comprehensive and forward-looking exploration of sustainable practices and innovative technologies in the field of wood-based materials and products. Several studies (Reh et al. and Silva et al.) focus on alternative raw materials and eco-friendly adhesive formulations, highlighting the potential of underutilized wood species and nanoparticle-enhanced resins to improve performance while reducing environmental impact. Complementary research (Reh et al., Gumowska et al.) advances the use of biopolymers, natural fillers, and bio-based additives, aiming to minimize formaldehyde emissions and develop fully biodegradable, formaldehyde-free composites.

The enhancement of material properties through physical and chemical modifications is another recurring theme. The contributions by Rahayu et al. and Jurczykova et al. demonstrate how impregnation and inorganic chemical treatments can improve wood’s durability, dimensional stability, and pollutant resistance, while thermal modification (Jurczykova et al.) offers improved structural properties at the expense of aesthetic value.

Specialized applications, such as artistic coatings (Han and Yan) and acoustic performance (Faktorová et al.), reveal the nuanced influence of surface treatments and additives on the visual, tactile, and auditory properties of wood, expanding its functional and creative potential. Finally, Pakula et al. contribute practical insights into the energy demands of furniture manufacturing, balancing environmental responsibility with user convenience and mechanical durability.

The articles collectively emphasize a growing commitment to sustainability, innovation, and efficiency within the wood-based materials industry. Across all contributions, there is a clear shift toward eco-friendly practices, whether through the use of alternative wood species, bio-based adhesives, or advanced material modifications.

Researchers are increasingly exploring ways to reduce environmental impact, enhance performance, and utilize natural or waste-derived materials more effectively. This includes the integration of nanoparticles, biopolymers, and plant-based additives, as well as process improvements such as thermal treatments and impregnation.

Another common theme is the importance of balancing functionality, aesthetics, and sustainability. Whether optimizing acoustic performance, durability, or manufacturing energy use, the studies demonstrate that thoughtful material selection and processing techniques can lead to better, greener products.

Overall, these contributions reflect a dynamic and evolving field, where science and technology are being applied to meet modern demands for environmentally responsible, high-performing wood-based materials across various applications—from construction and furniture to fine arts and acoustics.
